# Recent Progress in the Application of Electrospinning Technology in the Biomedical Field

**DOI:** 10.3390/jfb16070266

**Published:** 2025-07-18

**Authors:** Qun Wang, Peng Ji, Tian Bu, Yating Mao, Hailun He, Naijing Ge

**Affiliations:** 1 School of Chemistry, Chemical and Materials Engineering, Taizhou University, Taizhou 225300, China; wangqun@tzu.edu.cn; 2 School of Pharmacy, Taizhou University, Taizhou 225300, China

**Keywords:** electrospinning, nanofibers, biomedical materials, tissue engineering, drug delivery systems, mass production

## Abstract

Electrospinning has emerged as a highly effective technique for fabricating micro- and nanofibers, which are characterized by high porosity, large surface area, and structural mimicry of the extracellular matrix (ECM). These properties render it particularly suitable for biomedical applications. This review provides a comprehensive overview of recent developments in electrospinning-based strategies across various biomedical fields, including tissue engineering, drug delivery, wound healing, enzyme immobilization, biosensing, and protective materials. The distinctive advantages of electrospun fibers—such as excellent biocompatibility, tunable architecture, and facile surface functionalization—are discussed, alongside challenges such as the toxicity of organic solvents and limitations in scalability. Emerging approaches, including environmentally benign electrospinning techniques and integration with advanced technologies such as 3D printing and microfluidics, present promising solutions for intelligent and personalized biomedical applications.

## 1. Introduction

Electrospinning is a versatile and efficient technique for the fabrication of micro- and nanoscale fibers, offering distinct advantages such as high surface area, interconnected porosity, and tunable morphology. Since its initial development in the 1930s and subsequent resurgence in the late 20th century, electrospinning has garnered increasing interest in biomedical engineering due to its capacity to produce fiber architectures that closely mimic the structural and functional characteristics of the natural extracellular matrix (ECM). This biomimicry facilitates cellular adhesion, proliferation, and nutrient diffusion, positioning electrospun materials as promising candidates for a broad spectrum of biomedical applications, including wound healing, drug delivery, enzyme immobilization, tissue engineering, and biosensing [1,2,3,4,5].

Several review articles have addressed the development and biomedical applications of electrospinning. For instance, Chronakis [6] summarized the processing techniques and applications of electrospun fibers, emphasizing their structural similarity to ECM scaffolds. Pant et al. [7] focused on core-sheath nanofibers for drug delivery, highlighting the potential of coaxial electrospinning in achieving controlled release and multi-agent encapsulation. More recently, Liang et al. [8] reviewed advances in tendon–bone interface engineering using aligned electrospun fibers, underscoring the relevance of fiber orientation and biofunctionalization. While these studies collectively demonstrate the broad applicability of electrospun materials, challenges such as batch-to-batch variability, solvent toxicity, and scalability persist, limiting clinical translation.

Notably, most existing reviews concentrate on isolated application areas and rarely address the convergence of electrospinning with emerging technologies such as 3D bioprinting, microfluidics, or stimuli-responsive systems, which are advancements that are critical for the development of next-generation biomedical devices.

The present review aims to provide a comprehensive and up-to-date overview of electrospinning in biomedical applications, offering a systematic analysis of the underlying principles, material classifications, and key operational parameters. The discussion spans multiple application areas, including tissue engineering scaffolds, drug delivery systems (DDS), enzyme carriers, biosensors, and protective mask materials. Emphasis is placed on the unique functional attributes of electrospun fibers—such as bio-mimetic structural design, bioactive payload capacity, cellular modulation, and smart response—as well as on current limitations and future directions.

By integrating recent findings and interdisciplinary innovations, this review outlines the technological landscape and prospective advancements in electrospinning, with particular attention paid to multifunctional integration, sustainable processing, and translational potential. This synthesis aims to inform researchers and clinicians of the state-of-the-art methods and guide future efforts in developing intelligent and personalized biomedical solutions.

## 2. Overview of Electrospinning Technology

### 2.1. Principle of Electrospinning Technology

Electrospinning technology is a method capable of fabricating ultrafine fibers, and it currently represents the direct and continuous approach to preparing polymer nanofibers. It finds extensive application in organic and inorganic polymer solutions or melts [9], employing minimal flow rates to impart kilovolt tension to viscous mixtures, thereby transforming these fluids into fibers [10]. Fibers produced through electrospinning typically exhibit diameters spanning from tens of nanometers to tens of micrometers, characterized by high precision and uniformity. Additionally, the electrospinning fiber webs possess high porosity and large specific surface areas [11], along with properties such as high magnetic permeability, low basis weight, and the capacity to retain electrostatic charges [6]. In 1934, Formalas [12], while researching electrospinning technology, invented a device for preparing polymer fibers using this technique. The patent clearly elucidates the principle of converting solutions into fibers, marking it as the first detailed description of a device utilizing high-voltage electrostatics for fiber preparation, thereby heralding the advent of electrospinning fiber preparation technology.

**Figure 1:**
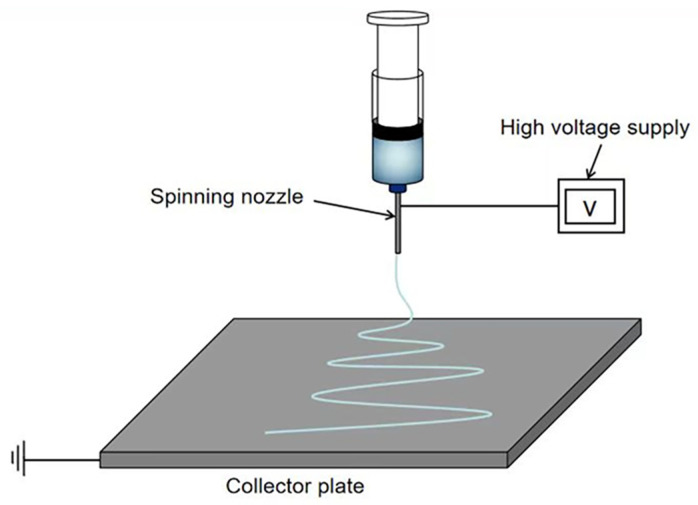
Schematic diagram of the vertical electrospinning device.

**Figure 2:**
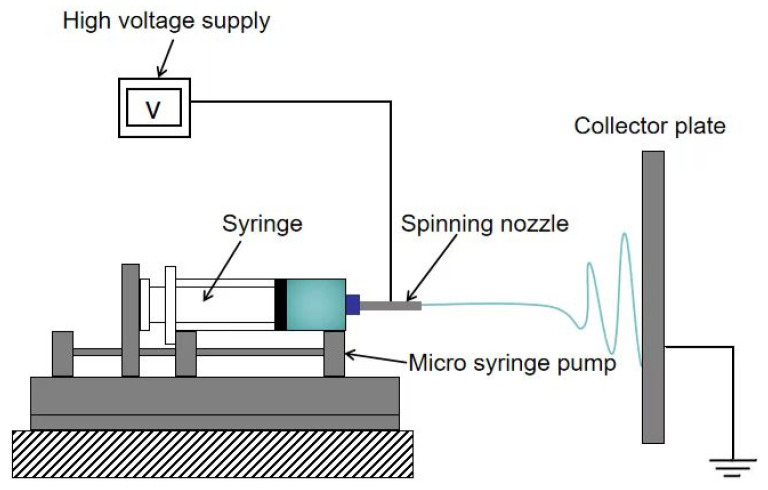
Schematic diagram of the horizontal electrospinning device.

The equipment for electrospinning typically comprises the following four main components: a high-voltage power supply, a collection device, a solution storage unit, and an ejection device, among others. The prevalent spinning techniques are vertical spinning [13] and horizontal spinning [14], as illustrated in Figure 1 and Figure 2, respectively. During the fabrication process, a high-voltage power supply, ranging from several thousand volts to several tens of thousands of volts, is employed to establish a voltage differential between the solution and the collection device. This enables the solution to overcome its surface tension and form a Taylor cone [15]. When the voltage reaches a critical level, the electrostatic force acting on the polymer solution or melt becomes sufficient to overcome both the surface tension and viscous resistance of the fluid. Consequently, a charged jet is ejected from the apex of the Taylor cone towards the cathode. This charged jet undergoes unstable bending and rapid oscillations in the air, causing it to stretch and thin out dramatically, resulting in ultrafine fibers that eventually deposit onto the surface of the collection device [16].

### 2.2. Classification of Electrospinning Technology

Electrospinning technology can be categorized into the following two types based on the distinct states of the polymers employed in the spinning process: melt electrospinning [17] and solution electrospinning [18].

Melt electrospinning has garnered significant attention in both academic and industrial circles, owing to its absence of toxic solvents and capacity for large-scale production. This technique involves heat transfer through a molten jet, facilitating a solvent-free and mass-transfer-exempt process that yields microfibers thicker than those produced via solution electrospinning, which typically results in nanofibers [19]. As a solvent-free process [20], melt electrospinning offers cost reduction, enhanced productivity, and environmental friendliness [21]. Notably, even in the absence of suitable solvents, melt electrospinning can produce highly uniform nanofibers with nearly identical fiber diameters [22]. While melt electrospinning offers a solvent-free alternative to traditional electrospinning methods, it is accompanied by several drawbacks that may limit its effectiveness and applicability. Challenges related to nanoscale fiber diameter, thermal degradation, limited polymer selection, equipment complexity, and jet stability, among others, underscore the necessity for ongoing investigation and innovation in this field [23,24,25,26,27]. Addressing these issues is crucial for unlocking the full potential of melt electrospinning in various applications, particularly in tissue engineering and DDS.

Solution electrospinning entails mixing organic solvents and raw materials in precise proportions to form a solution, which is then sprayed as a jet under high pressure. This jet undergoes stretching and oscillation in the air before being deposited onto a rotating collector, ultimately yielding nanofibers [28]. The process hinges on solvent mass transfer and evaporation to create ultrafine fibers. However, a major limitation in this method stems from the use of organic solvents, which are often volatile, toxic, and flammable, posing challenges in the preparation of nanofibers [29]. In addition, solution electrospinning encounters several challenges [30], including the need for a high-voltage power supply, susceptibility to needle tip clogging, and limited control over fiber arrangement, orientation, and structure. Furthermore, this technique is only applicable to charged polymer solutions and presents difficulties in scaling up for industrial production [31].

### 2.3. Factors Affecting Electrospinning

**Figure 3:**
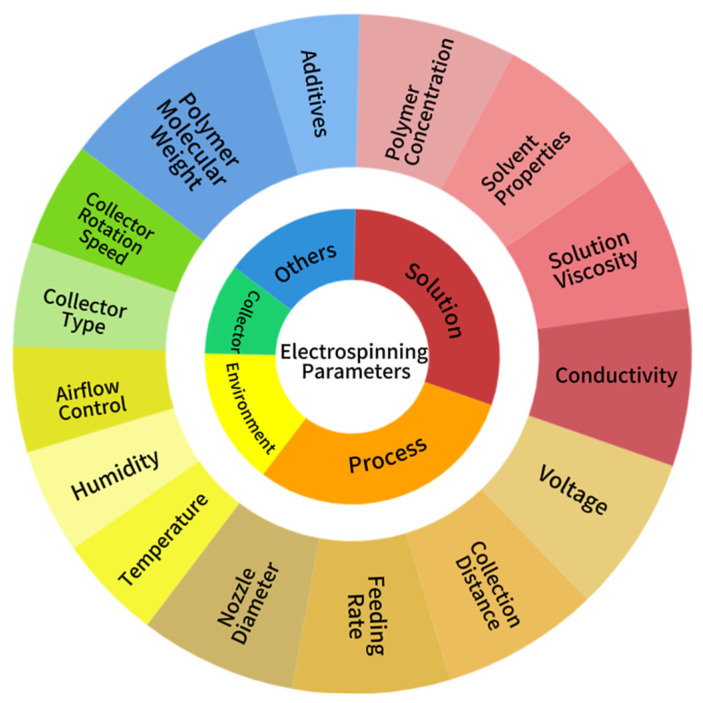
Factors that affect electrospinning.

As shown in Figure 3, there are numerous influencing factors involved in the preparation process of electrospinning [32,33,34], which can be broadly categorized into the following four groups: the properties of the solution or melt, spinning process factors, and environmental parameters. Specifically, the properties of the solution encompass concentration, solvent properties, viscosity, and conductivity. The spinning process factors include voltage level, collection distance, feed rate, and nozzle diameter. Environmental parameters, on the other hand, encompass temperature, humidity, and indoor airflow control. The selection of collector type also plays a critical role in determining fiber morphology and alignment characteristics. The following three primary collector configurations are commonly employed: (1) static flat plate collectors, which produce randomly oriented nonwoven fiber mats; (2) rotating drum collectors that enable the fabrication of aligned fiber architectures through mechanical stretching; and (3) specialized patterned collectors designed for creating three-dimensional fibrous structures. Particularly for rotating drum systems, the rotational velocity serves as a crucial processing parameter, where increased angular velocity induces greater fiber alignment by enhancing uniaxial tensile forces during fiber deposition.

Varying these parameters can significantly impact the properties of the nanofibers. By adjusting these parameters accordingly, nanofibers with diverse properties can be produced for application in various fields.

## 3. Overview of Biomedical Materials

### 3.1. Introduction to Biomedical Materials

Biomedical materials, specifically designed for medical applications, are a category of specialized materials characterized by their unique properties and functions. They are utilized for treating injuries, restoring the functionality of human tissues or organs, and diagnosing or treating diseases, all without posing any harm to the human body. Typically, they encompass tissue engineering materials [8], wound healing dressings [35], drug delivery vector materials [36], immobilized enzyme materials [37], biosensors [38], and more. Consequently, advancements in biomedical materials contribute significantly to medical progress and enhance people’s quality of life.

Based on the level of biochemical reactions they undergo in physiological environments, biomedical materials are categorized into inert, active, degradable, and absorbable biomedical materials.

Furthermore, as shown in Table 1 [39,40,41,42,43,44,45,46,47,48,49,50,51,52,53], depending on their composition and properties, they are classified into biomedical metal, ceramics, polymer materials, composite materials, and bioderived materials [54].

### 3.2. Requirements of Electrospun Biomedical Materials

Biomedical materials prepared through electrospinning technology, such as artificial organs and wound dressings, serve as substitutes for damaged organs and tissues, significantly enhancing the success rate of treatment. However, to avoid adverse biological reactions during clinical application, medical supplies must adhere to the following three fundamental principles: Firstly, they must exhibit excellent biocompatibility, meaning that, upon contact with the body, they should not elicit inflammation, foreign body reactions, or coagulation. Secondly, the materials must possess stable chemical properties, characterized by high corrosion resistance and non-reactivity with the body’s metabolic biochemistry or enzyme catalysis, ensuring that their performance remains consistent over time. Thirdly, they must possess suitable mechanical properties. Depending on their application in different parts of the human body, materials may require high strength and toughness, or they may need to be elastic. Additionally, the requirements for the shape, structure, and absorption properties of medical materials vary accordingly.

When utilizing electrospinning technology to produce medical supplies, raw material selection, process design, and quality control should all adhere to these three principles.

## 4. Application of Electrospinning Technology in the Preparation of Biomaterials

In recent years, significant advancements have been achieved in the research of electrospinning technology within the realm of biomedical materials. The micro–nano fibers fabricated via this technology possess distinctive features such as unique structural biomimicry, high specific surface area, and functionalizable properties. Consequently, they have found extensive applications in various fields, including tissue engineering materials, drug delivery carriers, biosensors, enzyme immobilization carriers, and medical mask materials.

### 4.1. Tissue Engineering Scaffolds

Tissue engineering scaffolds serve as alternative tissues for the transplantation, reconstruction, and repair of human organs and tissues, with the pivotal component being the “biological scaffold” [55]. Its primary role is to procure adequate nutrients from tissues to sustain cellular life and to facilitate the regular removal of metabolic waste. The scaffolds must possess certain qualities, including high porosity, a large specific surface area, adjustable structural strength, non-immunogenicity [56], and excellent biocompatibility, among others. Thanks to its numerous advantageous properties, electrospinning technology enables the regeneration and treatment of target tissues and finds widespread application in areas such as bone, cartilage, blood vessels, skin tissue, and neural tissue.

#### 4.1.1. Bone Tissue Engineering

Bone injuries and defects resulting from congenital malformations, accidents, tumors, and other factors can impair the normal physiological functions of the human body. With the growing proportion of the elderly population, the incidence of degenerative bone diseases has also increased. Hence, there is an urgent need to address such therapeutic challenges.

Wu et al. [57] blended a copolymer of lactide and caprolactone (PLCL) with gelatin (Gel) or collagen (Col) and fabricated a scaffold membrane through electrospinning technology (voltage: 16–18 kV; receiving distance: 17 cm; liquid supply rate: 3 mL/h; spinning time: 2 h; spinning temperature: 25 °C). This membrane was then cultured with mouse cranial pre-osteoblastic cell lines, followed by MTS colorimetric and cell mineralization assessments. Both PLCL/Gel and PLCL/Col composite membranes exhibited the ability to enhance cell adhesion and proliferation, demonstrating a certain osteogenic induction effect.

Fu et al. [58] prepared radial bioplastic poly(3-hydroxybutyrate-co-4-hydroxybutyrate) (P34HB) electrospun fiber scaffolds. The morphology of stem cells on these scaffolds post-culture was observed under a fluorescence microscope, and cell viability assessments were conducted. The results indicated that the P34HB electrospun fiber scaffolds could enhance the locking and fixation between implants and bones.

Peidavosi et al. [59] fabricated piezoelectric responsive and conductive nanofiber series comprising binary polycaprolactone/barium titanate (PCL/BT), PCL/polyaniline (PANI), and ternary PCL/PANI/BT composites using electrospinning technology, followed by MTT cytotoxicity tests. All samples exhibited non-toxicity, with cell growth and proliferation observed. These composite fibers serve as active platforms for cell growth and proliferation, presenting themselves as promising substitutes for bone tissue engineering scaffolds.

Massoumi et al. [60] employed electrospinning technology to fabricate novel conductive nanofiber scaffolds comprising chitosan-grafted polythiophene (CTS-g-PTh) and a blend (3/1, *w*/*w*) of chitosan-grafted polythiophene (CTS-g-PTh) with polycaprolactone (PCL). Their findings revealed that, within the same timeframe, the CTS-g-PTh/PCL scaffolds exhibited a slightly higher cell proliferation rate, enhanced nanofiber uniformity, and superior performance. These studies collectively underscore the potential of nanofibers prepared via electrospinning with diverse raw materials for applications in bone tissue engineering.

#### 4.1.2. Cartilage Tissue Engineering

Cartilage tissue is a type of avascular connective tissue characterized by its slow metabolism, healing process, and regeneration capacity, necessitating the use of appropriate biomaterial scaffolds to facilitate tissue regeneration and repair [61]. Electrospun nanofiber scaffolds, mimicking the fibrous structure of the extracellular matrix, have garnered considerable attention in research focused on cartilage tissue repair.

Sadat et al. [62] incorporated 5 wt.% and 7 wt.% of glucosamine sulfate (GAS) into a poly(3-hydroxybutyrate)-chitosan (PHB-CTS) composite solution, utilizing GAS as the natural extracellular matrix (ECM) of cartilage tissue, to fabricate nanofibers. These nanofibers were evaluated in terms of their mechanical properties, adipose-derived stem cell proliferation, and stem cell differentiation. The study revealed that the incorporation of GAS enhanced the hydrophilicity of the fibers and the extent of cartilage differentiation.

Xu et al. [63] developed a nanofiber scaffold through the electrospinning of a composite solution, which contained a total solute concentration of 10% and maintained a weight ratio of PCL to gelatin at 1:1. Six New Zealand white rabbits were selected for subcutaneous implantation of sterilized nanofiber scaffolds along their dorsal midline, while another six rabbits served as the control group without any material implantation. At eight weeks post-implantation, cellular proliferation was observed around the implanted material, forming distinct fibrous capsule walls, suggesting that the scaffold facilitated cell growth.

**Figure 4:**
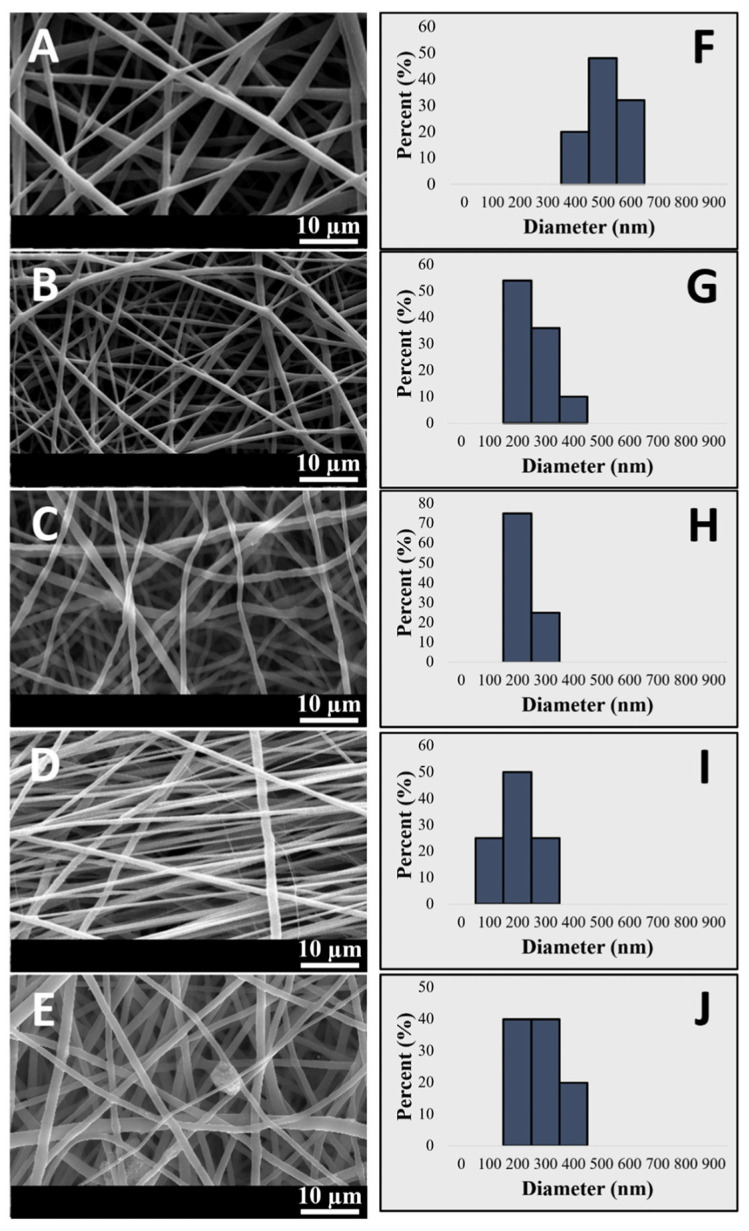
SEM micrographs and fiber diameter histogram of PHB (**A**,**F**); PHB-starch (**B**,**G**); 0.5 wt.% (**C**,**H**), 2 wt.% (**D**,**I**), and 2.5 wt.% (**E**,**J**) of incorporated HNTs. Reproduced with permission from Ref. [64], Copyright 2022, Elsevier B.V.

Movahedi et al. [64] prepared a fiber scaffold composed of hydrophobic PHB and hydrophilic starch via electrospinning technology, reinforced with halloysite nanotubes (HNTs). SEM imaging (as shown in Figure 4) and MTT assays demonstrated that the nanofibers incorporating HNTs exhibited smaller diameters and larger pores. Furthermore, HNTs supported cell growth and attachment, rendering them suitable for cartilage tissue applications.

Implanting biomaterials capable of controlling the release of antibacterial agents during articular cartilage repair can prevent postoperative infections. Samie et al. [65] fabricated a biomimetic non-woven nanofiber scaffold loaded with cefixime trihydrate (CFX), incorporating varying proportions of PCL, polylactic acid (PLA), and silk fibroin (SF) through the use of electrospinning technology. By adjusting the formulation and processing parameters, the release profile of the drug can be tailored. This scaffold exhibits antibacterial activity against *Staphylococcus aureus* and *Escherichia coli* under static in vitro conditions. When cocultured with the NIH-3T3 fibroblast cell line, the drug-loaded nanofiber mat demonstrated comparable cell compatibility to pure polycaprolactone nanofibers.

#### 4.1.3. Vascular Tissue Engineering

Currently, cardiovascular and cerebrovascular diseases are increasingly prevalent in our daily lives. When confronted with these conditions, drug treatment often proves ineffective, necessitating vascular graft surgery. While large-diameter vascular stents have been extensively utilized in clinical settings, small-diameter vascular stents (with a diameter of less than 6 mm) are still under investigation. This is due to their stringent requirements in terms of blood compatibility [66] and mechanical strength, as well as the challenges associated with post-transplantation issues such as thrombosis, inflammation, and stent occlusion [67].

Chernonosova et al. [68] fabricated vascular grafts with a diameter of 80.1 mm through electrospinning technology by blending polyurethane with gelatin or bivalirudin. Their research revealed that the vascular prosthesis generated in small animal models (mice and rats) exhibited superior blood and biocompatibility.

Hosseinzadeh et al. [69] devised a substrate capable of controlling the thickness of blood vessel walls. By mixing gelatin with growth hormone and utilizing electrospinning technology, they created fiber scaffolds. Upon comparing mechanical strength, cell proliferation, and release kinetics, they discovered that the integration of growth hormone into nanofibers resulted in a sustained-release effect, thereby enhancing the proliferation of vascular tissue cells.

Wu et al. [70] incorporated heparin into the materials used for preparing vascular stents and implanted these fiber stents into the femoral artery defects of dogs. Through angiography, it was observed that the sustained release of heparin exhibited anticoagulant therapeutic effects.

#### 4.1.4. Skin Tissue Engineering

The skin serves as a tissue and organ in direct contact with the external environment of the human body. When damaged, it can result in impaired barrier function and trigger infections. Traditional wound dressings exhibit weak antibacterial and anti-infective properties and are prone to adhering to the wound surface [71]. Removing these dressings can cause pain and inadvertently tear off newly formed skin, hindering the recovery process. There are two conventional methods for skin defect transplantation, autologous skin transplantation and xenotransplantation [72].

However, both methods have their respective drawbacks. Autologous skin transplantation may lead to uneven skin texture, necrosis, and insufficient skin grafts for large-scale transplantation. On the other hand, xenotransplantation can elicit rejection reactions, causing swelling, systemic fever, and lymphocyte proliferation as the patient’s body does not adapt to the foreign tissue. Electrospun fabrics boast high porosity and excellent air permeability. The raw materials used for their preparation are abundant, enabling the fabrication of nanofibers from gelatin, CTS, and polyurethane, among others [73]. The wound dressings produced from these fibers exhibit excellent air permeability, strong absorptive capacity, robust antibacterial properties, and effective hemostasis [74].

Ranjbarvan et al. [75] utilized polysaccharides and nylon 66 as raw materials to create highly efficient composite nanofibers. Following cell seeding, real-time PCR technology was employed to monitor epidermal gene expression. Immunohistochemical analysis revealed that the fiber scaffolds preserved the inherent properties of keratinocytes and exhibited elastic characteristics akin to natural skin. Agnes Mary et al. [76] discovered that the hydrophobicity of polycaprolactone hindered its application in tissue engineering materials. Consequently, the contact angle of PCL nanofiber mat incorporating 15% (by mass) aloe extract was reduced by 61° (from 89° to 28°), compared to those without aloe extract, indicating an increase in hydrophilicity, thus ultimately resulting in a faster proliferation rate of fibroblasts. These enhanced nanofibers hold potential for use in skin tissue dressings. Norouzi et al. [77] encapsulated epidermal growth factor within polylactic acid–glycolic acid copolymer (PLGA) nanofibers and assessed the proliferation of human fibroblasts. Their findings indicated that the scaffold released epidermal growth factor at a desired rate while maintaining excellent biocompatibility, rendering it suitable for applications in skin tissue engineering.

Li et al. [78] leveraged the potent antibacterial activity of CTS, along with the hemostatic capabilities of collagen, to develop nanofiber-based dressings, thus discovering that when the CTS ratio exceeded 65%, the nanofibers exhibited enhanced antibacterial efficacy against *Escherichia coli* and *Staphylococcus aureus*. Cui et al. [79] utilized PVA-styrene pyridinium salt and zein as raw materials to fabricate composite nanofibers with varying mass ratios through electrospinning technology. Flavonoid glycosides were loaded onto these fibers. When compared with Vaseline gauze, the nanofiber membrane served as a control. It was observed that Vaseline gauze adhered to wounds, potentially causing secondary damage upon removal due to tearing. In contrast, the nanofiber membrane possessed degradability and gradually detached from the skin over time.

#### 4.1.5. Dental Implants Scaffolds

The application of electrospun scaffolds in dental implants is supported by various studies highlighting their potential benefits. For instance, Xu et al. demonstrated that electrospun nanofibers can mimic the ECM, which is crucial for cell attachment and proliferation, thereby enhancing tissue integration around dental implants [80]. Similarly, Mathur et al. reported that electrospun gelatin scaffolds embedded with silver nanoparticles exhibited significant antimicrobial properties, promoting soft tissue attachment and reducing the risk of infections around dental implants [81]. Furthermore, the study by V et al. indicated that a nanocomposite fibrous scaffold could enhance osseointegration in rabbit mandibular defects, suggesting that electrospun scaffolds can effectively promote new bone formation and integration with dental implants [82]. These findings collectively underscore the ability of electrospun scaffolds to improve the biological performance of dental implants through enhanced cell interaction and tissue regeneration.

Despite the promising applications of electrospun scaffolds in dental implants, there are notable concerns regarding their effectiveness. For example, while the study by Anitua et al. highlighted the benefits of using growth factor-rich scaffolds for bone regeneration, it also pointed out that the success of such scaffolds can be variable and dependent on the specific biological environment [83]. Additionally, the research by Kavoosi et al. emphasized that, while electrospun scaffolds can enhance cell adhesion, they may also present challenges such as low mechanical strength and potential inflammatory responses due to degradation products [84]. Moreover, the study by Liu et al. indicated that the integration of electrospun scaffolds with existing bone structures can be inconsistent, leading to variable outcomes in clinical applications [85]. These limitations suggest that, while electrospun scaffolds have potential, their practical application in dental implants may face significant hurdles.

#### 4.1.6. Summary

Electrospinning technology exhibits vast application potential in the field of tissue engineering, as it enables the fabrication of nanofiber scaffolds characterized by high porosity, a large specific surface area, and a biomimetic ECM structure. Presently, a wide array of biocompatible polymers and their composites have been successfully converted into nanofiber scaffolds using electrospinning technology, finding applications in tissue engineering for bones, cartilage, blood vessels, skin, and other tissues. This underscores the remarkable advantages of electrospun scaffolds in terms of biomimetic structural design, bioactive payload capacity, and cellular modulation.

### 4.2. Drug Delivery Carriers

Currently, a variety of DDS have emerged in the medical field, enhancing the therapeutic efficacy of drugs while mitigating their toxicity and side effects. The ideal attributes of DDS encompass high drug loading capacity, low cost, ease of operation, and robust controllability of drug release rate, all of which can be achieved through the preparation of nanofibers via electrospinning technology. Nanofibers can be fabricated using a wide range of raw materials, including large and small molecule drugs, as well as various other materials, enabling them to exhibit pharmacological effects while also offering controlled release capabilities [86]. The majority of research on nanofiber-based DDS focuses on stimulus-responsive systems, where drug release behavior is contingent upon changes in environmental parameters such as pH, temperature, and magnetic fields. By altering the raw material mass ratio or preparation parameters, electrospinning technology allows for the adjustment of the drug release rate in nanofiber DDS.

#### 4.2.1. Anti-Infective Drug Delivery System Using Electrospun Nanofibers

When the body’s immune system is weakened, it becomes susceptible to microbial infections, including bacteria, mycoplasmas, viruses, and parasites. The most prevalent treatment approach is the administration of antibiotics. However, excessive reliance on antibiotics can readily enhance microbial resistance. Consequently, there is a pressing need to develop an efficient antibiotic delivery system that targets the site of infection, thereby preventing overmedication. Electrospinning technology allows for the fabrication of nanofiber materials loaded with anti-infective drugs, exhibiting remarkable anti-infective efficacy, as illustrated in Table 2.

#### 4.2.2. Electrostatically Spun Nanofiber Drug Delivery System with Anti-Inflammatory Properties

Inflammation is a defensive response of the body to various stimuli, which is generally beneficial; however, if not effectively controlled, it can lead to serious consequences such as fever, toxic shock, and hypotension. Anti-inflammatory medications primarily include non-steroidal anti-inflammatory drugs and steroidal anti-inflammatory drugs. Ideally, these agents provide rapid symptomatic relief. Consequently, many researchers have incorporated anti-inflammatory drugs into nanofibers to investigate their drug release rates and anti-inflammatory effects. Some examples are included below.

Kamath et al. [99] prepared ibuprofen-loaded PCL nanofiber interlayers within a sandwich-structured fiber mat. Various drug-loaded nanofiber mats were fabricated by altering the concentration of PCL in the core layer. The study’s findings indicated that the crystallinity and hydrophobicity of the core fibers significantly influenced the release behavior of ibuprofen, while no chemical reaction occurred between ibuprofen and PCL fibers, ensuring that the drug’s efficacy remained unaffected.

He et al. [100] incorporated ibuprofen into PLGA/PLA composite nanofibers to develop guided tissue regeneration membranes for periodontal surgery. In vitro drug release tests demonstrated that there was no abrupt release of ibuprofen; additionally, the drug-carrying fibrous membrane system exhibited excellent biocompatibility, effective barrier function, and a slow-release profile.

Immich et al. [101] investigated drug release from PCL sandwich membranes produced via an electrostatic spinning process. Their research revealed that the mechanisms governing drug release behaviors were primarily determined by membrane thickness rather than drug concentration.

Su et al. [102] developed aspirin (AS)/corn starch (ST)/polyvinyl alcohol (PVA) composite nanofiber membranes using electrostatic spinning technology. Results from in vitro dissolution experiments indicated that the controlled release of AS could be achieved by adjusting both its loading amount and the ratio of PVA to ST.

#### 4.2.3. Anti-Tumor Class Electrospun Nanofiber Drug Delivery System

Currently, the predominant modalities for cancer treatment are chemotherapy and radiotherapy. Chemotherapy typically involves the administration of cytotoxic agents aimed at inhibiting the proliferation of cancer cells; however, this approach can result in significant adverse reactions. Consequently, a more desirable therapeutic strategy is to achieve targeted drug delivery that primarily affects tumor tissues while sparing normal cells. Examples are included in Table 3.

#### 4.2.4. Intelligent Response Electrospun Nanofiber DDS

The advancement of responsive systems capable of modulating drug delivery in accordance with the microscopic environment, by integrating bio-responsive and environment-sensitive properties into nanofibers, facilitates targeted drug therapy. For instance, as shown in Table 4, pH-responsive and temperature-sensitive smart fibers can release drugs under specific conditions, thereby enhancing therapeutic efficacy while minimizing toxic side effects.

(i). pH-stimulated response drug delivery system

Salehi et al. [109] developed nanofibers incorporating doxorubicin (DOX) through the electrospinning of pH-sensitive N-isopropylacrylamide-methacrylic acid-vinyl pyrrolidone (P(NIPAAm-MAA-VP)). This system achieved a drug loading capacity exceeding 90%, allowing for the controlled release of DOX at physiological pH levels ranging from 5.4 to 7.4.

Jiang et al. [110], leveraging the overexpression of hyaluronic acid (HA) receptors on the surface of most malignant tumor cells, demonstrated that HA could serve as both a micellar carrier for protecting DOX and a ligand for targeted therapy in tumor cells. Consequently, they constructed hyaluronic acid–hydrazone bond–adriamycin hydrochloride nanofibers, wherein the hydrazone bond was cleaved at approximately pH = 5, facilitating the release of adriamycin hydrochloride and thereby achieving targeted therapeutic effects.

(ii). Temperature-stimulated response drug delivery system

Toan et al. [111] and colleagues developed composite electrospun nanofibers incorporating the drug ibuprofen, utilizing polycaprolactone (PCL), a temperature-responsive polymer known as N-isopropylacrylamide (PNIPAM), and a PNIPAM/PCL blend. Their findings indicated that the drug-loaded nanofibers derived from the PNIPAM/PCL combination exhibited the controlled release of ibuprofen at temperatures 25 °C and 34 °C.

Elashnikov et al. [112] synthesized poly-L-propylene carbonate/PNIPAM temperature-responsive nanofibers containing crystalline violet in varying ratios. They discovered that modulating the ambient temperature above the lower critical solution temperature (32 °C) of PNIPAM effectively facilitated control over crystalline violet release.

Zhou et al. [113] incorporated curcumin and polydopamine nanoparticles (PDANPs) into polycaprolactone (PCL) nanofibers. The study revealed that PDANPs possess photothermal conversion properties; when the temperature reaches a specific threshold, they can induce tumor cell death and facilitate the release of curcumin. This process not only inhibits tumor cell growth but also promotes the clearance of these cells.

Guo et al. [114] utilized free radical polymerization to prepare poly (hydroxyethyl acrylate-coumarate acrylate-ethyl methacrylate) (P(HEA-CA-EMA)) as thermos-responsive nanofibers through an electrospinning technique. The UV-treated P(HEA-CA-EMA) nanofibers demonstrated the controlled release of the encapsulated 5(6)-carboxyfluorescein at temperatures exceeding the lowest critical solubilization temperature, thereby achieving a regulated drug release profile.

Zheng et al. [115] synthesized a copolymer of N-isopropylacrylamide (NIPAM) and N-hydroxymethacrylamide, subsequently fabricating nanofibers through electrospinning. These fibers were then dispersed in tert-butyl alcohol via mechanical cutting and shortening, followed by freeze-drying and heat treatment to produce a porous nanofiber hydrogel. The study revealed that this type of nanofiber hydrogel exhibited a rapid temperature response when the water temperature alternated between 20 °C and 55 °C. Additionally, its loading capacity was enhanced during temperature fluctuations from 15 °C to 47 °C. Notably, it was observed that the drug-loaded dextran was released in an “on/off” manner as the temperature varied between 15 °C and 47 °C.

#### 4.2.5. Summary

Electrostatic spinning technology can modify the factors that influence the preparation process, thereby enabling drug-loaded nanofibers to effectively control drug release. In the context of anti-infection, given the limitations in developing new antibiotics and to prevent the human body from acquiring resistance to these drugs, it is essential to develop nanofibers that can deliver medication directly at designated infection sites. This targeted approach minimizes hydrolysis in non-infected tissues and preserves therapeutic efficacy. Additionally, certain hydrophobic anti-inflammatory drugs can be incorporated into nanofibers, broadening their application potential. Furthermore, while anti-tumor drugs exhibit significant inhibitory effects on normal cells, employing controlled DDS facilitates targeted treatment strategies that aim to reduce the side effects associated with cancer therapies.

The application of electrostatic spinning technology in smart-responsive DDS offers significant advantages, particularly in the development of pH-responsive and temperature-responsive nanofibers. In pH-responsive systems, nanofibers constructed from pH-sensitive polymers (e.g., P(NIPAAm-MAA-VP)) or targeting ligands (e.g., hyaluronic acid-perylene bond) can precisely release drugs (e.g., doxorubicin) within the tumor microenvironment (pH 5.4~7.4), thereby facilitating efficient targeted therapy. Temperature-responsive systems integrate temperature-sensitive materials such as PNIPAM and PCL with photothermal agents (e.g., polydopamine nanorods) to achieve controlled drug release (e.g., ibuprofen, curcumin) at specific temperatures. These systems can also be combined with photothermal therapy to enhance anti-tumor effects. Such smart responsive systems not only improve the spatial and temporal controllability of drug delivery but also significantly mitigate toxic side effects, thus providing innovative approaches for personalized therapy. However, optimizing material stability and response sensitivity and scaling up production processes remain critical focuses for future research endeavors.

### 4.3. Enzyme Immobilization Materials

The stability of enzymes is significantly influenced by environmental factors, including variations in pH, temperature, and the presence of organic solvents. These factors can lead to enzyme denaturation and subsequent inactivation. Consequently, the advent of immobilized enzyme technology has provided solutions to these challenges. Electrospun nanofiber membranes possess intricate structures and are readily recyclable. Numerous researchers have utilized these nanofibers for the immobilization of enzymes, and some of these examples are shown below.

Zheng et al. [116] employed electrospinning technology to fabricate nanofibers of styrene maleic anhydride copolymer, which were subsequently utilized for the immobilization of β-D-galactosidase. It was observed that the immobilized enzyme retained 85% of its initial activity after 21 consecutive operations at 37 °C while catalyzing the hydrolysis reaction of 2-nitrophenol-β-D-galactopyranoside. Furthermore, when applied in a continuously stirred reactor for lactose hydrolysis, the immobilized enzyme demonstrated stable operation over a period of 17 consecutive days.

Sui et al. [117] developed polyacrylic acid/PVA fiber membranes using coupling agents and activators, successfully immobilizing glucose amylase via an activated ester method. After a storage duration of 12 days, the activity of the immobilized enzyme was found to be 62% relative to that of the fresh enzyme; additionally, it maintained an activity level of 40.5% even after being used repeatedly for ten cycles.

Xu et al. [118] prepared PVA and glucose oxidase as components for immobilized enzymes, which exhibited sustained activity within electrospun nanofibers and demonstrated superior current response characteristics compared to those obtained through traditional casting membrane methods. The electrochemical response properties of these enzyme electrodes were further enhanced by incorporating nano gold into the electrospinning solution.

Silva et al. [119] successfully immobilized trypsin onto electrospun nanofibers utilizing PET and PLA as carriers; they discovered that this enzymatic preparation could be stored in water at 4 °C for a minimum duration of 30 days without loss in activity upon reuse up to eight times, with no observable leaching occurring during this process.

Therefore, electrospinning technology can effectively preserve enzyme activity and enhance enzyme stability. The application of electrospun nanofibers for enzyme immobilization does not compromise their structural integrity, and a variety of raw materials are available for both the immobilization of enzymes and the specific modification of enzymes to meet diverse environmental requirements. This approach is anticipated to improve the efficiency of enzyme utilization while simultaneously reducing costs.

### 4.4. Biosensor Materials

Biosensors offer significant advantages in various fields, including environmental monitoring, clinical medicine, and health assessment. Consequently, their utilization has become widespread. Human sweat and saliva contain a diverse array of enzymes that provide valuable information reflecting the body’s health status. When discomfort arises within the body, these biosensors can promptly and accurately indicate unusual conditions. Therefore, it is essential for biosensors to possess high sensitivity and stability [120]. Electrospun nanofibers exhibit a large specific surface area, enabling them to accommodate more biological elements and significantly enhance the sensitivity of biosensors, and some examples are shown below.

Li et al. [121] prepared zinc oxide precursors through electrospinning, followed by calcination to produce zinc oxide micro–nanofibers. They utilized the regenerated zinc oxide fibers to immobilize tyrosinase, which enhanced stability and improved sensitivity in catechol detection. Xu et al. [122] incorporated carbon nanotubes and magnetic nanoparticles into electrospun fibers to develop electrochemical impedance sensors and electroluminescent immunosensors, respectively. These sensors were employed for monitoring formaldehyde, alpha-fetoprotein, and aflatoxin B1. The sensors fabricated by leveraging the advantages of nanofibers and nanoparticles demonstrated rapid detection capabilities, high sensitivity, and excellent stability.

Zhou et al. [123] employed coaxial electrospinning to create a three-dimensional porous composite nanoelectrode consisting of zinc oxide and copper oxide, significantly enhancing its sensitivity for glucose monitoring. Jing et al. utilized a spinning solution composed of Prussian blue, CTS, and PVA to fabricate nanofibers that were subsequently electrodeposited into chemical sensors. These sensors exhibited remarkable stability in glucose monitoring without interference from coexisting substances, thereby facilitating further research on sensor applications for proteins, enzymes, and other analytes.

Wu et al. [124,125] loaded hemoglobin onto gold nanoparticle-CTS-PVA composite nanofibers for hydrogen peroxide monitoring; they found that hemoglobin not only retained its electrochemical activity but also enhanced monitoring sensitivity along with high selectivity and reproducibility, among other benefits.

The primary requirements for biosensors are high sensitivity and excellent selectivity. Nanofibers produced through electrospinning possess the advantage of high porosity, which aligns well with the demands of biosensor applications. By incorporating various raw materials, it is possible to fabricate specific nanofibers that can be extensively utilized for monitoring a range of target components.

### 4.5. Application in Mask Filter Materials

After more than three years of the COVID-19 pandemic, masks have become an essential part of daily life. The protective efficacy of a mask is contingent upon the filter material utilized. Conventional filter materials typically possess large fiber diameters and pore sizes, lacking antibacterial properties. Consequently, they are unable to effectively block certain bacteria and are susceptible to cross-contamination and secondary pollution. In contrast, electrospun nanofibers exhibit diameters in the nanometer range and can be infused with antibacterial agents. These nanofibers not only provide efficient filtration against viruses and bacteria but also demonstrate sterilizing effects [126].

Kangle et al. [127] investigated the impact of electrospun polyvinylidene fluoride (PVDF) nanofiber membranes on filtration performance across various processing conditions. Through a comparative analysis of multiple spinning techniques, it was determined that the PVDF nanofiber membrane exhibits high filtration efficiency, low filtration resistance, and an extended service life when produced at a concentration of 15%, with an applied voltage of 27 kV, a distance of 22 cm, and a flow rate of 2.5 mL/h. Furthermore, upon incorporating silver nanoparticles into the membrane structure, it was observed that the antibacterial efficacy of the PVDF nanofiber membrane can exceed 99%.

Baselga et al. [128] utilized trifluoroacetic acid and dichloromethane as solvents, employing recycled PET as the raw material to produce ultrafine fibers via electrospinning technology. These ultrafine fibers were incorporated into the filter element layer of a three-layer medical surgical mask, which demonstrated filtration efficiency exceeding 98.2% for particles with diameters ranging from 0.5 to 10 μm.

The research objectives for masks extend beyond merely achieving small fiber diameters, high porosity, and superior filtration efficiency. There is also a significant interest in utilizing electrospinning technology to incorporate antibacterial agents into nanofibers, thereby endowing masks with antibacterial properties. Currently, studies have demonstrated the feasibility of loading certain antibacterial drugs onto fibers to confer antibacterial effects [129]; however, these electrospun antibacterial masks are not yet suitable for mass production and cannot be commercially available at this time.

In summary, electrospinning technology is propelling the evolution of masks towards enhanced efficiency, intelligence, and environmental sustainability. In the future, it may progressively supplant traditional melt-blown fabrics as the predominant filtering material, particularly in high-end protective applications and specialized fields.

## 5. Conclusions and Outlook

### 5.1. Conclusions

Electrospinning technology, recognized as an advanced method for the efficient preparation of nanofibers, has demonstrated significant potential for applications in the field of biomedical materials in recent years. The resulting nanofibers exhibit several advantages, including a small diameter, high porosity, large specific surface area, and robust biomimetic structures. These characteristics enable them to closely mimic the topological structure of natural ECM, thereby providing an optimal environment for cell growth, nutrient transport, and metabolic waste disposal.

Furthermore, electrospinning technology is characterized by its wide adaptability to various raw materials, controllable morphology, excellent biocompatibility, and ease of functional modification. These attributes contribute to its extensive utilization in diverse areas, such as tissue engineering, DDS, wound dressings, enzyme immobilization techniques, biosensors development, and medical protective materials. This research progress is illustrated in Table 5.

### 5.2. Challenges and Opportunities

Despite the significant advancements achieved in electrospinning technology, several challenges and opportunities remain to be addressed.

Firstly, the challenges are as follows:

Large-scale production: Currently, electrospinning technology is predominantly at the laboratory research stage, and significant difficulties remain in achieving the large-scale production of electrospun nanofibers. During the preparation process, errors may occur in formulating the polymer spinning solution. Additionally, numerous factors influence electrospinning technology, making it challenging to consistently control both the diameter and quality of nanofibers over extended periods. It is recommended to optimize process parameters—such as voltage, temperature, and humidity control—to enhance fiber uniformity and yield.

Environmental protection and safety: Although a variety of polymers have been identified for use as spinning solutions in nanofiber preparation, most spinning solvents exhibit certain levels of toxicity that pose risks to personnel involved in their handling. Furthermore, these solvents can adversely affect the environment and hinder efforts toward sustainable production practices. It is advisable to minimize reliance on organic solvents (for instance, by promoting melt electrospinning) while also assessing the biocompatibility of materials intended for long-term implantation.

Nevertheless, with advancements in technology, ongoing research progress, and continuous equipment upgrades, these challenges are expected to be addressed effectively. This will facilitate the transition of electrospinning technology from laboratory settings to industrialization and commercialization stages.

Secondly, the opportunities are listed as follows:

Multifunctional integration: The emphasis should be placed on the development of composite fibers that integrate mechanical strength, biological activity, and intelligent responsiveness to address complex clinical requirements.

Interdisciplinary integration: Technologies such as 3D printing and microfluidics should be synergistically combined to construct biomimetic multi-level structures that facilitate advancements in personalized medicine.

In summary, electrospinning technology has emerged as one of the core methodologies within the field of biomaterials due to its unique capacity for structural design and functional diversity. With the deepening integration of material science and medicine, its application prospects in regenerative medicine, precision therapy, and intelligent medical devices are poised to expand significantly.

## Figures and Tables

**Table 1:** Types and examples of different class biomaterials.

Types of Biomaterials	Examples	References
Biomedical Metals	Stainless Steel (316L); Titanium(Ti-6Al-4V)	[39,40,41]
Bioceramics	Hydroxyapatite (HA); Zirconia (ZrO_2_)	[42,43,44]
Biopolymer Materials	Poly(lactic-co-glycolic acid) (PLGA); CTS	[45,46,47]
Biocomposite Materials	HA/Chitosan Composites; PEEK/Carbon Fiber Composites	[48,49,50]
Bio-derived Materials	Decellularized Extracellular Matrix (dECM); Silk Fibroin	[51,52,53]

**Table 2:** Typical representatives of electrospinning technology in anti-infective DDS.

Antibacterial Active Ingredients	Carrier of Active Ingredients	Product Form	Performance	References
Nano silver	PET nanofiber	membrane	Good antibacterial and anti-biofilm activity against Gram positive bacteria, Gram negative bacteria, and fungi, with reduced cytotoxicity and weakened inflammatory effects.	[87]
Metronidazole (MNA)	PCL nanofiber	membrane	The loaded MNA can be released in a controlled manner, and the sustained release time can exceed two weeks.	[88]
Nano-silver, Vancomycin	Collagen nanofiber	membrane	The bactericidal effect on *Escherichia coli* and *Staphylococcus aureus* was better than the samples loaded with a single antimicrobial drug component.	[89]
2% Chlorhexidine	PVA nanofiber	membrane	It has a significant antibacterial effect on *Candida albicans* and *E. faecalis* in the oral cavity.	[90]
Ciprofloxacin Hydrochloride and Gentamicin Sulfate	Gelatin nanofiber	scaffold	Improved antimicrobial properties of gelatin scaffolds against *Pseudomonas aeruginosa* and *Staphylococcus aureus*.	[91]
Rifampicin, Vancomycin, Linezolid, Daptomycin	PCL, PLGA nanofiber composite	coating	Effective in preventing bone/joint tissue infections and biofilm formation on implants.	[92]
Naringenin	PCL, PEG nanofiber	mat	Sustained release of Naringenin at high concentrations of Nrg; comparable re-epithelialization and wound closure effects to commercially available phenytoin sodium ointment.	[93]
Ciprofloxacin	PVA, CTS, SF nanofiber	mat	The prepared PChS mats showed bactericidal activity against *Escherichia coli* and *Staphylococcus aureus*.	[94]
Tannic acid	CTS, PVA nanofiber	sponge	High porosity, high water absorption and retention capacity, high hemostatic capacity, high antimicrobial and antioxidant capacity, showing high biocompatibility to L929 cells and ability to accelerate wound healing.	[95]
*Syzygium cumini* leaf extract (SCLE)	PLGA, PMMA nanofiber	mat	Nanofiber mats containing concentrations of 0.5% and 1% (*w*/*v*) of SCLE showed better antimicrobial effects than pure extracts of the same concentration.	[96]
Natural propolis	Microcrystalline Cellulose, CTS nanofiber	wound dressing	Good cytocompatibility, showing significant bactericidal effects against both Gram positive and negative bacteria.	[97]
Antimicrobial peptide CM11	SF nanofiber	SF fiber/amniotic membrane bilayer composite	The drug-loaded complexes inhibited bacterial growth, the wound closure rate was significantly higher in wounds covered by bilayer complexes loaded with 32 μg/mL of CM11, and the relative expression levels of collagen type I, collagen type III, TGF-β1, and TGF-β3 were higher than those in other groups.	[98]

**Table 3:** Typical representatives of electrospinning technology in anti-tumor DDS.

Anticancer Active Ingredients	Carrier of Active Ingredients	Product Form	Performance	References
Cisplatin	PLA nanofibers	nanofibrous mats	In contrast to the pristine PLA nanofibers, those incorporated with 0.2 wt.% cisplatin exhibited significant inhibitory effects on oral squamous cell carcinoma cell (CAL-27) proliferation in vitro.	[103]
Paclitaxel and Cisplatin	Polypropylene carbonate fiber	fibrous membrane	Significant inhibition of A549 lung adenocarcinoma cell proliferation was observed in vitro.	[104]
Sirolimus	Core-shell structured nanofibers composed of a PCL core and a CTS/PCL composite shell	nanofibrous mats	Core-shell nanofibers showed sustained release (>480 h), outperforming uniaxial fibers. They suppressed MKI67, MMP-2/9 in glioma cells in vitro, and exhibited superior antitumor efficacy in vivo (smaller tumors, increased necrosis, no toxicity) versus drug suspensions.	[105]
Curcumin and 5-fluorouracil	Regenerated SF/PEG composite nanofibers	fibrous membrane	The electrospinning process preserved the secondary structure of silk fibroin, while the co-encapsulated dual ingredients exhibited sustained release profiles (400 h) from the nanofibrous membranes.	[106]
Sodium dichloroacetate	PLA nanofibers	nanofibrous mats	Significant antitumor efficacy was observed, achieving a 94% tumor inhibition rate (*p* < 0.05) and a 38% complete remission rate in tumor-bearing mice.	[107]
Resveratrol	mPEG-PCL block copolymer nanofibers	nanofibrous mats	In vitro XTT assays demonstrated significantly enhanced cytotoxicity of resveratrol-loaded nanofibrous scaffolds (RSV NFS) against U87 glioma cells. Compared to equivalent doses of free resveratrol, RSV NFS treatment resulted in markedly reduced clonogenic capacity, impaired cell migration, and suppressed invasive potential.	[108]

**Table 4:** Representatives of different response types of DDS.

Active Ingredients	Carrier of Active Ingredients	Response Type	Performance	References
DOX	P(NIPAAm-MAA-VP) nanofiber	pH	Achieved a drug loading capacity exceeding 90%, allowing for the controlled release of DOX at physiological pH levels ranging from 5.4 to 7.4.	[109]
Adriamycin hydrochloride	hydrazone bonded HA nanofiber		The hydrazone bond was cleaved at approximately pH = 5, facilitating the release of adriamycin hydrochloride and thereby achieving targeted therapeutic effects.	[110]
Ibuprofen	PNIPAM/PCL blend	Temperature	The drug-loaded nanofibers derived from the PNIPAM/PCL combination exhibited the controlled release of ibuprofen at temperatures of 25 °C and 34 °C.	[111]
Crystalline violet	poly-L-propylene carbonate/PNIPAM nanofiber		Modulating the ambient temperature above the lower critical solution temperature (32 °C) of PNIPAM effectively facilitated control over crystalline violet release.	[112]
Curcumin and PDANPs	PCL nanofiber		PDANPs possess photothermal conversion properties; when the temperature reaches a specific threshold, they can induce tumor cell death and facilitate the release of curcumin. This process not only inhibits tumor cell growth but also promotes the clearance of these cells.	[113]
5(6)-carboxyfluorescein	P(HEA-CA-EMA) nanofiber		This study demonstrated the controlled release of the encapsulated 5(6)-carboxyfluorescein at temperatures exceeding the lowest critical solubilization temperature, thereby achieving a regulated drug release profile.	[114]
Dextran	copolymer of (NIPAM) and N-hydroxymethacrylamide nanofiber		This type of nanofiber hydrogel exhibited a rapid temperature response when the water temperature alternated between 20 °C and 55 °C. Additionally, its loading capacity was enhanced during temperature fluctuations from 15 °C to 47 °C. Notably, it was observed that the drug-loaded dextran was released in an “on/off” manner as the temperature varied between 15 °C and 47 °C.	[115]

**Table 5:** Research progress of electrospinning technology in different fields.

Fields	Research Progress
Tissue Engineering	Bone tissue: enhances cellular activity and promotes both cell proliferation and differentiation. Blood vessels: improve blood compatibility and facilitate endothelialization. Skin: accelerates healing processes and combats infections.
Drug Delivery	The nano scaffold materials generated through electrospinning technology can be incorporated with a variety of drugs. By modulating the fiber composition and structure, it is possible to achieve controlled drug release, thereby enhancing the therapeutic efficacy.
Enzyme Immobilization	The elevated specific surface area and the modifiable nature of electrospun nanofibers offer stable platforms for enzyme immobilization, thereby significantly enhancing both the reusability and stability of enzymes.
Biosensor	The nanofiber structure can be functionalized with basic affinity agents, such as enzymes and proteins, for use in biosensors. This configuration enables the detection of target substances, including glucose and hydrogen peroxide, with high sensitivity. Consequently, it facilitates the advancement of real-time diagnostic technologies.
Face Mask	Electrospun fiber masks exhibit high filtration efficiency while maintaining the smoothness of human respiration, thereby enhancing user comfort. Additionally, these masks possess antibacterial properties. It is anticipated that they will replace traditional melt-blown fabrics and facilitate the advancement of protective equipment towards greater efficiency and intelligence.

## Data Availability

The original contributions presented in this study are included in the article. Further inquiries can be directed to the corresponding author(s).

## Abbreviations

The following abbreviations are used in this manuscript:

ECM	extracellular matrix
DDS	drug delivery systems
PLCL	copolymer of lactide and caprolactone
Gel	gelatin
Col	collagen
P34HB	poly(3-hydroxybutyrate-co-4-hydroxybutyrate)
PANI	polyaniline
PCL	polycaprolactone
BT	barium titanate
CTS-g-PTh	chitosan-grafted polythiophene
PHB-CTS	poly(3-hydroxybutyrate)-chitosan
GAS	glucosamine sulfate
HNTs	halloysite nanotubes
CFX	cefixime trihydrate
PLA	polylactic acid
SF	silk fibroin
PCR	polymerase chain reaction
PLGA	polylactic acid-glycolic acid copolymer
PET	polyethylene glycol terephthalate
MNA	metronidazole
PVA	polyvinyl alcohol
CTS	chitosan
SCLE	syzygium cumini leaf extract
PChS	polyvinyl alcohol-chitosan
AS	aspirin
ST	corn starch
DOX	doxorubicin
P(NIPAAm-MAA-VP)	N-isopropylacrylamide-methacrylic acid-vinyl pyrrolidone
PDANPs	polydopamine nanoparticles
HA	hyaluronic acid
PNIPAM	N-isopropylacrylamide
P(HEA-CA-EMA)	poly(hydroxyethyl acrylate-coumarate acrylate-ethyl methacrylate)
NIPAM	N-isopropylacrylamide
PVDF	polyvinylidene fluoride
MMP	matrix metalloproteinases
MKI67	proliferation marker protein Ki-67
PEG	polyethylene glycol
RSV NFS	resveratrol-loaded nanofibrous scaffolds

## References

[1] Berdimurodov E., Dagdag O., Berdimuradov K., Rbaa M., Safin F.O., El Ibrahimi B. (2023). Green Electrospun Nanofibers for Biomedicine and Biotechnology. Technologies.

[2] Liu S., Yu J., Gan Y., Qiao Y., Wu S., Zhao W., Wang Y., Zhou Q. (2024). Biomimetic natural biomaterials for tissue engineering and regenerative medicine: New biosynthesis methods, recent advances, and emerging applications. Mil. Med. Res..

[3] Wang J., You C., Xu Y., Liu T., Zhang B., Li F. (2024). Research Advances in Electrospun Nanofiber Membranes for Non-Invasive Medical Applications. Micromachines.

[4] Saba F., Hadi M.N., Siavash I. (2023). Nanophotocatalysts in biomedicine: Cancer therapeutic, tissue engineering, biosensing, and drug delivery applications. Environ. Res..

[5] Maran V.A.B., Jeyachandran S., Kimura M. (2024). A Review on the Electrospinning of Polymer Nanofibers and Its Biomedical Applications. J. Compos. Sci..

[6] Chronakis I.S. (2010). Micro-/nano-fibers by electrospinning technology: Processing, properties and applications. Micro Manuf. Eng. Technol..

[7] Pant B., Park M., Park S.J. (2019). Drug Delivery Applications of Core-Sheath Nanofibers Prepared by Coaxial Electrospinning: A Review. Pharmaceutics.

[8] Liang C., Fan Z., Zhang Z., Wang P., Deng H., Tao J. (2024). Electrospinning technology: A promising approach for tendon-bone interface tissue engineering. RSC Adv..

[9] Ren S.S., Miao L.Y., Sun W.B. (2017). Research Progress of Near-Field Electrospinning Technology in Biological Fields. Oral Biomed..

[10] Bulu E., Sakarya G., Altndal T., Yaman E., Ayten N.Y., Kamacı Ö., Akkaş M. Effects and Characterization of Electrospinning Technique Working Parameters on Polymeric Membrane Morphology. Proceedings of the International Agricultural, Biological & Life Science Conference.

[11] Zhao X.H., Liu G.Y., Duan C.M. (2016). Application of Electrospun Fibers in Biomedical Field. Text. Sci. Technol. Prog..

[12] Formhals A. (1934). Process and Apparatus for Preparing Artificial Threads. U.S. Patent.

[13] Liu C.K. (2012). Research Progress of Electrospinning Technology. Synth. Fiber Ind..

[14] Zhang Y.L. (2015). Optimization of Rotary Cup Electrospinning Device and Process.

[15] Guan S., Gai G.Q. (2022). Application of Electrospinning Technology in Various Fields. Technol. Dev. Chem. Ind..

[16] Wang Y.Z. (2018). Brief History and Applications of Electrospinning Technology. Synth. Fiber Ind..

[17] Manea L.R., Bertea A., Popa A., Hristian L., Bertea V.P. (2018). Melt Electrospinning–Characteristics, Application Areas and Perspectives. IOP Conf. Ser. Mater. Sci. Eng..

[18] Wang Y. (2015). Study on Electrospinning of Water-Soluble Polymer Solutions.

[19] Bachs-Herrera A., Yousefzade O., Del Valle Núñez L.J., Puiggalí J., Alshamsi A.A. (2021). Melt electrospinning of polymers: Blends, nanocomposites, additives and applications. Appl. Sci..

[20] Li X.Y., Liu J.L., Li C.J. (2012). Application of melt electrospinning technology in tissue Engineering. New Chem. Mater..

[21] Huang T., Marshall L.R., Armantrout J.E., Yembrick S., Dunn W.H., Oconnor J.M., Mueller T., Avgousti M., Wetzel M.D. (2012). Production of Nanofibers by Melt Spinning. U.S. Patent.

[22] Dayan C.B., Afghah F., Okan B.S., Yildiz O., Menceloglu Y.Z. (2018). Modeling 3D melt electrospinning writing by response surface methodology. Mater. Des..

[23] Zhou H., Green T.B., Joo Y.L. (2006). The thermal effects on electrospinning of polylactic acid melts. Polymer.

[24] Dalton P.D., Grafahrend D., Klinkhammer K., Klee D., Möller M. (2007). Electrospinning of polymer melts: Phenomenological observations. Polymer.

[25] Hutmacher D.W., Dalton P.D. (2011). Melt electrospinning. Chem. Asian J..

[26] Deng R., Liu Y., Ding Y., Zhu M. (2009). Melt electrospinning of low-density polyethylene having a low-melt flow index. J. Appl. Polym. Sci..

[27] Hochleitner G., Youssef A., Hrynevich A., Dalton P.D., Groll J. (2016). Fibre pulsing during melt electrospinning writing. BioNanoMaterials.

[28] Chen J., Zhang D.J., Zhang T.J., Zhang M.L., Wang C. (2018). Preparation of thermoplastic polyimide ultrafine fiber nonwovens by solution electrospinning. J. Mater. Eng..

[29] Fang N.Z., Feng H., Dai L.X. (2009). Effects of freezing treatment on electrospinning of a-PVA/s-PVA blend solution. Synth. Fiber Ind..

[30] Song J., Kim M., Lee H. (2020). Recent advances on nanofiber fabrications: Unconventional state-of-the-art spinning techniques. Polymers.

[31] Mogharbel R.T., Almahri A., Alaysuy O., Al-Lohedan H.A., Elaasser M.M., Bakhsh E.M., Alzamel N.Z. (2023). Preparation of photochromic solution blow spun polycarbonate nanofibers from recycled plastic for optical anticounterfeiting. Opt. Mater..

[32] Dai L.Q., Zhang R.Q. (2013). Application and Development Trends of Electrospinning Technology. J. Wuhan Text. Univ..

[33] Liu Y.S. (2016). Study on Preparation and Properties of Functional Fibers by Electrospinning.

[34] Blechta V. (2022). Permeable Membranes PUR/TETA and PUR/TEPA for CO_2_ Capture Prepared with One-Step Electrospinning Technology. Fibers.

[35] Cheng L., Gao Y., Zhou J., Xu Y., Zhang J. (2011). Classification and Features of Medical Dressings. China Med. Devices.

[36] Renukadevi C.R., Ayyanaar S., Kesavan M.P., Dhas T., Sivagnanam U.T. (2023). Reactive oxygen species responsive magnetic polylactic co-glycolic acid microspheres: In vitro drug release studies. Mater. Today Commun..

[37] Shaowei D., Allison A.C., Igor L.M., Rotello V.M. (2015). Increasing the activity of immobilized enzymes with nanoparticle conjugation. Curr. Opin. Biotechnol..

[38] Chao J., Zhu D., Zhang Y., Wang L., Fan C. (2016). DNA nanotechnology-enabled biosensors. Biosens. Bioelectron..

[39] Niinomi M. (2008). Mechanical biocompatibilities of titanium alloys for biomedical applications. J. Mech. Behav. Biomed. Mater..

[40] Geetha M., Singh A.K., Asokamani R., Gogia A.K. (2009). Ti based biomaterials, the ultimate choice for orthopaedic implants—A review. Prog. Mater. Sci..

[41] Asad M., Sana M. (2023). Potential of titanium based alloys in the biomedical sector and their surface modification techniques: A review. Proc. Inst. Mech. Eng. C.

[42] LeGeros R.Z. (2002). Properties of osteoconductive biomaterials: Calcium phosphates. Clin. Orthop. Relat. Res..

[43] Piconi C., Maccauro G. (1999). Zirconia as a ceramic biomaterial. Biomaterials.

[44] Kurtz S.M., Kocagoz S., Arnholt C., Huet R., Ueno M. (2014). Advances in zirconia toughened alumina biomaterials for total joint replacement. J. Mech. Behav. Biomed. Mater..

[45] Jain R.A. (2000). The manufacturing techniques of various drug-loaded biodegradable poly(lactide-co-glycolide) (PLGA) devices. Biomaterials.

[46] Rinaudo M. (2006). Chitin and chitosan: Properties and applications. Prog. Polym. Sci..

[47] Manasa M.T., Ramanamurthy K.V., Arun Bhupathi P. (2023). Electrospun nanofibrous wound dressings: A review on chitosan composite nanofibers as potential wound dressings. Int. J. Appl. Pharm..

[48] Thein-Han W.W., Misra R.D.K. (2009). Biomimetic chitosan–nanohydroxyapatite composite scaffolds for bone tissue engineering. Acta Biomater..

[49] Kurtz S.M., Devine J.N. (2007). PEEK biomaterials in trauma, orthopedic, and spinal implants. Biomaterials.

[50] Verma S., Sharma N., Kango S., Singh R., Saini V.K., Thakur V.K. (2021). Developments of PEEK (polyetheretherketone) as a biomedical material: A focused review. Eur. Polym. J..

[51] Badylak S.F. (2007). The extracellular matrix as a biologic scaffold material. Biomaterials.

[52] Altman G.H., Diaz F., Jakuba C., Calabro T., Horan R.L., Chen J., Lu H.H., Richmond J., Kaplan D.L. (2003). Silk-based biomaterials. Biomaterials.

[53] Yadav R.H., Kenchegowda M., Angolkar M., Singh R., Bansal S., Yadav S.K. (2024). A review of silk fibroin-based drug delivery systems and their applications. Eur. Polym. J..

[54] Guo L.L. (2021). Construction of Biofunctional Coatings on Biomedical Materials and Their Antibacterial/Antifouling Properties.

[55] Zhang T.F., Liu Z.Y., Zhang Y., Wang L., Li J., Zhao X. (2022). Research Status of Materials and Preparation Methods for Tissue Engineering Scaffolds. China Rubber Plast. Technol. Equip..

[56] Song J.K. (2012). Preparation of Cellulose Nanofibers and Their Application in Tissue Engineering Scaffolds.

[57] Wu G.L., Ren T.B., Cao C.H., Li Y., Zhang L., Liu J., Wang X. (2010). Preparation of PLCL/gelatin and PLCL/collagen guided bone tissue regeneration membranes by electrospinning and their structure, properties and cytology. Mater. Rev..

[58] Fu N., Meng Z., Jiao T., Li Y., Wang Y., Zhang L., Liu C. (2020). Radial P34HB Electrospun Fiber: A Scaffold for Bone Tissue Engineering. J. Nanosci. Nanotechnol..

[59] Peidavosi N., Azami M., Beheshtizadeh N., Ebrahimi-Barough S., Saber-Samandari S., Akbari J., Mozafari M. (2022). Piezoelectric conductive electrospun nanocomposite PCL/Polyaniline/Barium Titanate scaffold for tissue engineering applications. Sci. Rep..

[60] Massoumi B., Abbasian M., Khalilzadeh B., Fathi M., Farajzadeh M.A., Aghajani H., Davoodi J. (2021). Electrically Conductive Nanofibers Composed of Chitosan-grafted Polythiophene and Poly(ε-caprolactone) as Tissue Engineering Scaffold. Fibers Polym..

[61] Stoddart M.J., Della B.E., Armiento A.R. (2023). Cartilage Tissue Engineering: An Introduction. Methods Mol. Biol..

[62] Sadat N.G., Saeed K., Elahe M., Rezaei M., Farajzadeh M., Jafari S.M., Akbari J. (2022). Evaluation of the effects of glucosamine sulfate on poly(3-hydroxybutyrate)-chitosan/carbon nanotubes electrospun scaffold for cartilage tissue engineering applications. Polym.-Plast. Technol. Mater..

[63] Xu Z.L., Liu J., Zhang C.Q. (2013). Construction of a cartilage tissue engineering scaffold by electrospun polycaprolactone-gelatin nanofiber membranes combined with rabbit bone marrow mesenchymal stem cells. Int. J. Orthop..

[64] Movahedi M., Karbasi S. (2022). Electrospun halloysite nanotube loaded polyhydroxybutyrate-starch fibers for cartilage tissue engineering. Int. J. Biol. Macromol..

[65] Samie M., Khan A.F., Hardy J.G., Saeb M.R., Mozafari M., Annabi N. (2022). Electrospun antibacterial composites for cartilage tissue engineering. Macromol. Biosci..

[66] Zhou W., Feng Y.K., Yang J., Fu J.X., Lv J., Zhang L., Guo J.T., Ren X.K., Zhang W.C. (2015). Electrospun scaffolds of silk fibroin and poly(lactide-co-glycolide) for endothelial cell growth. J. Mater. Sci. Mater. Med..

[67] Huang C. (2013). Preparation of Electrospun Tubular Scaffolds and Their Application in Tissue Engineering.

[68] Chernonosova V., Gostev A., Murashov I., Krivandin A., Shishatskiy S., Filatov A. (2021). Assessment of Electrospun Pellethane-Based Scaffolds for Vascular Tissue Engineering. Materials.

[69] Hosseinzadeh S., Zarei-Behjani Z., Bohlouli M., Khorasani M., Akbari J., Mozafari M. (2021). Fabrication and optimization of bioactive cylindrical scaffold prepared by electrospinning for vascular tissue engineering. Iran. Polym. J..

[70] Wu T. (2014). Electrospun Protein-Polysaccharide-Poly(lactide-co-caprolactone) Composite Nanofiber Scaffolds for Small-Diameter Vascular Tissue Engineering.

[71] Su L.A., Leng X.F., He C., Zhang Y., Li J., Wang X. (2022). Effect of a novel electrospun nano-scaffold dressing on rat skin wound. J. Qingdao Univ. Med. Sci..

[72] Lei Y., Zhang L.F. (2013). Experimental study on electrospun PLGA-collagen scaffold for skin tissue engineering. J. Biomed. Eng. Res..

[73] Li A., Li X.L. (2022). Research progress on electrospun nanofiber skin wound dressings. China J. Lepr. Skin. Dis..

[74] Li H., Cheng W., Liu K., Zhang Y., Wang L., Zhao X., Chen J. (2017). Reinforced collagen with oxidized microcrystalline cellulose shows improved hemostatic effects. Carbohydr. Polym..

[75] Ranjbarvan P., Mahmoudifard M., Kehtari M., Saber-Samandari S., Akbari J., Mozafari M. (2018). Natural compounds for skin tissue engineering by electrospinning of nylon-Beta vulgaris. ASAIO J..

[76] Agnes Mary S., Giri Dev V.R. (2015). Electrospun herbal nanofibrous wound dressings for skin tissue engineering. J. Text. Inst..

[77] Norouzi M., Shabani I., Atyabi F., Dorkoosh F.A., Akbari J. (2015). EGF-loaded nanofibrous scaffold for skin tissue engineering applications. Fibers Polym..

[78] Li X. (2017). Preparation and Characterization of Collagen/Chitosan Composite Antibacterial Dressing Membranes.

[79] Cui J. (2015). Electrospun PVA-SbQ/zein Drug-Loaded Nanofibers for Wound Healing Materials.

[80] Xu C., Inai R., Kotaki M., Ramakrishna S. (2004). Electrospun nanofiber fabrication as synthetic extracellular matrix and its potential for vascular tissue engineering. Tissue Eng..

[81] Mathur A., Kharbanda O.P., Koul V., Singh T., Gambhir R.S. (2022). Fabrication and evaluation of antimicrobial biomimetic nanofiber coating for improved dental implant bioseal: An in vitro study. J. Periodontol..

[82] Manju V., Iyer S., Menon D., Nair S.V., Jayakumar R. (2019). Evaluation of osseointegration of staged or simultaneously placed dental implants with nanocomposite fibrous scaffolds in rabbit mandibular defect. Mater. Sci. Eng. C.

[83] Anitua E., Orive G., Pla R., Andia I., Padilla S., Aguirre J. (2009). The effects of PRGF on bone regeneration and on titanium implant osseointegration in goats: A histologic and histomorphometric study. J. Biomed. Mater. Res. A.

[84] Safari F., Houshmand B., Nejad A.E. (2019). Application of zeolite, a biomaterial agent, in dental science: A review article. Regen. Reconstr. Restor..

[85] Liu W., Jiao T., Su Y. (2022). Electrospun porous poly (3-hydroxybutyrate-co-4-hydroxybutyrate)/lecithin scaffold for bone tissue engineering. RSC Adv..

[86] Torres-Martínez E.J., Cornejo Bravo J.M., Serrano Medina A., García-García A., Alvarez-Lorenzo C. (2018). A Summary of Electrospun Nanofibers as Drug Delivery System: Drugs Loaded and Biopolymers Used as Matrices. Curr. Drug Deliv..

[87] Grumezescu A.M., Stoica A.E., Dima-Bălcescu M.Ș., Mogoantă L., Andronescu E. (2019). Electrospun Polyethylene Terephthalate Nanofibers Loaded with Silver Nanoparticles: Novel Approach in Anti-Infective Therapy. J. Clin. Med..

[88] Xue J., He M., Niu Y., Zhang Y., Li J., Wang X. (2014). Preparation and in vivo efficient anti-infection property of GTR/GBR implant made by metronidazole loaded electrospun polycaprolactone nanofiber membrane. Int. J. Pharm..

[89] Lambert J., Ferrer J.S.J., Cornejo C.I. (2018). Antimicrobial electrospun collagen nanofibers loaded with Silver nanoparticles and Vancomycin. Proceedings of the Annual Meeting of The Japanese Pharmacological Society WCP2018 (The 18th World Congress of Basic and Clinical Pharmacology).

[90] Vaishali A., Varma K.M., Bhupathi P.A., Reddy S.K. (2017). In vitro evaluation of antimicrobial efficacy of 2% chlorhexidine loaded electrospun nanofibers. J. Pierre Fauchard Acad..

[91] Virijević K., Živanović M., Pavić J., Marković Z., Jovanović S. (2024). Electrospun Gelatin Scaffolds with Incorporated Antibiotics for Skin Wound Healing. Pharmaceuticals.

[92] Ashbaugh A.G., Jiang X., Zheng J., Anderson D.G., Bryers J.D. (2016). Polymeric nanofiber coating with tunable combinatorial antibiotic delivery prevents biofilm-associated infection in vivo. Proc. Natl. Acad. Sci. USA.

[93] Farzaei M.H., Derayat P., Pourmanouchehri Z., Shirazi M., Abdollahi M. (2023). Characterization and Evaluation of Antibacterial and Wound Healing Activity of Naringenin-Loaded Polyethylene Glycol/Polycaprolactone Electrospun Nanofibers. J. Drug Deliv. Sci. Technol..

[94] Kazemi M.H., Sajadimajd S., Karaji Z.G., Jafari S.M., Akbari J. (2023). In Vitro Investigation of Wound Healing Performance of PVA/Chitosan/Silk Electrospun Mat Loaded with Deferoxamine and Ciprofloxacin. Int. J. Biol. Macromol..

[95] Huang Z., Wang D., Sønderskov S.M., Chen X., Li Y., Yang F. (2023). Tannic Acid-Functionalized 3D Porous Nanofiber Sponge for Antibiotic-Free Wound Healing with Enhanced Hemostasis, Antibacterial, and Antioxidant Properties. J. Nanobiotechnol..

[96] Abdelazim E.B., Abed T., Goher S.S., El-Sayed N., El-Sherbiny I.M. (2024). In Vitro and in Vivo Studies of Syzygium Cumini-Loaded Electrospun PLGA/PMMA/Collagen Nanofibers for Accelerating Topical Wound Healing. RSC Adv..

[97] El-Ghoul Y., Altuwayjiri A.S., Alharbi G.A. (2024). Synthesis and Characterization of New Electrospun Medical Scaffold-Based Modified Cellulose Nanofiber and Bioactive Natural Propolis for Potential Wound Dressing Applications. RSC Adv..

[98] Mehrdad M.M., Behrouz F., Reza M., Saeedi M., Atyabi F. (2023). Evaluation of an antibacterial peptide-loaded amniotic membrane/silk fibroin electrospun nanofiber in wound healing. Int. Wound J..

[99] Kamath S.M., Sridhar K., Jaison D., Mathew A.K., Nair S.V., Jayakumar R. (2020). Fabrication of tri-layered electrospun polycaprolactone mats with improved sustained drug release profile. Sci. Rep..

[100] He M., Zhang E., Wu Z.Z., Li J., Wang Y., Chen H. (2014). Experimental Study on Ibuprofen-Loaded PLGA/PLA Electrospun Nanofibrous GTR Membranes. Chin. J. Conserv. Dent..

[101] Immich A.P.S., Arias M.L., Carreras N., Rubira A.F., Muniz E.C. (2013). Drug delivery systems using sandwich configurations of electrospun poly(lactic acid) nanofiber membranes and ibuprofen. Mater. Sci. Eng. C.

[102] Su Y.J., Chen G., Tang S.S., Zhang H., Liu J., Zhao X. (2015). Preparation and sustained-release properties of aspirin/starch/polyvinyl alcohol nanofiber membranes. J. Mol. Sci..

[103] Hao X., Hao D., Yang Z., Li Y., Wang L., Chen J. (2016). Preparation of Cisplatin-Containing Nanofibrous Mats and It’s In Vitro Antitumor Activity Against Oral Squamous Cell Carcinoma. J. Nanosci. Nanotechnol..

[104] Cui Y., Liu M.L., Wu B.Q., Zhang L., Zhao X., Wang Q. (2014). Inhibitory effect of combined paclitaxel-cisplatin drug delivery system on the growth of lung adenocarcinoma cell line A549. J. Cap. Med. Univ..

[105] Talimi R., Shahsavari Z., Dadashzadeh S., Mohammadi M., Akbari J., Mozafari M. (2023). Sirolimus-Exuding Core-Shell Nanofibers as an Implantable Carrier for Breast Cancer Therapy: Preparation, Characterization, In Vitro Cell Studies, and In Vivo Anti-Tumor Activity. Drug Dev. Ind. Pharm..

[106] Xie X., Yu J., Zhao Z., Wang L., Li J., Chen G. (2019). Fabrication and drug release properties of curcumin-loaded silk fibroin nanofibrous membranes. Adsorpt. Sci. Technol..

[107] Liu D.X., Wang M.Z., Jing X.B., Zhang Y., Li X., Zhao J. (2014). Sodium dichloroacetate electrospun fibrous mats improve the quality of life in mice with cervical cancer. Chin. J. Appl. Chem..

[108] Zhou H., Liu X., Wu F., Chen Y., Zhang L., Wang X. (2016). Preparation, Characterization, and Antitumor Evaluation of Electrospun Resveratrol Loaded Nanofibers. J. Nanomater..

[109] Salehi R., Irani M., Eskandani M., Khorasani M., Akbari J., Mozafari M. (2014). Interaction, Controlled Release, and Antitumor Activity of Doxorubicin Hydrochloride From pH-Sensitive P(NIPAAm-MAA-VP) Nanofibrous Scaffolds Prepared by Green Electrospinning. Int. J. Polym. Mater. Polym. Biomater..

[110] Jiang L.T. (2018). Construction and Performance Study of pH-Sensitive Virus-Mimetic Drug Delivery System.

[111] Tran T., Hernandez M., Patel D., Nguyen T.D., Lee S.W., Kim S.H. (2015). Controllable and switchable drug delivery of ibuprofen from temperature responsive composite nanofibers. Nano Converg..

[112] Elashnikov R., Slepička P., Rimpelova S., Sedlarik V., Kizek R. (2017). Temperature-responsive PLLA/PNIPAM nanofibers for switchable release. Mater. Sci. Eng. C.

[113] Zhou H.Y. (2021). Application of Polydopamine-Functionalized Nanofibers in Local Drug Delivery and Their Antitumor Performance.

[114] Guo H., Jeong J.H., Kim J.C. (2016). Electrospun thermo-responsive nanofibers of poly(hydroxyethylacrylate-co-coumaryl acrylate-co-ethylmethacrylate). Colloids Surf. A.

[115] Zheng X., Zha L.S. (2020). Preparation of ultrafast temperature-responsive nanofibrous hydrogels and their application in controlled drug release. Chin. J. Mater. Res..

[116] Zheng G.J., Zhang S.P., Zhang Y.F. (2010). Preparation of styrene-maleic anhydride copolymer nanofibers via electrospinning and their application in β-D-galactosidase immobilization. Chin. J. Process Eng..

[117] Sui C.H., Wang Z.Y., Wei Y.Q., Li M., Zhang J., Zhao Y. (2016). Immobilization of glucoamylase on PAA/PVA fibrous membrane by activated ester method. J. Process Eng..

[118] Xu X.H., Ren G.L., Liu Q., Zhang L., Wang X., Chen J. (2006). Immobilization of glucose oxidase by polyvinyl alcohol electrospinning. J. Tianjin Univ..

[119] Silva T.R., Rodrigues D.P., Rocha J.M., Malcata F.X., Pinto M.E. (2015). Immobilization of trypsin onto poly(ethylene terephthalate)/poly(lactic acid) nonwoven nanofiber mats. Biochem. Eng. J..

[120] Ji G., Chen Z., Li H., Wang Y., Zhang L., Liu J. (2022). Electrospinning-Based Biosensors for Health Monitoring. Biosensors.

[121] Li C., Sun L.X., Sun J.H., Zhao Y., Chen L., Wang Q. (2020). Preparation of ZnO by electrospinning for the construction of a tyrosinase biosensor to detect catechol. Chem. Ind. Eng. Prog..

[122] Xu G.F. (2016). Construction and Application of Electrospun Biosensors.

[123] Zhou C.Y. (2017). Research on Nanomaterial-Based Biosensors and Biomolecular Logic Devices.

[124] Wu J.P., Yin F. (2013). Sensitive enzymatic glucose biosensor fabricated by electrospinning composite nanofibers and electrodepositing Prussian blue film. J. Electroanal. Chem..

[125] Wu J.P., Yin F. (2013). Novel Hydrogen Peroxide Biosensor Based on Hemoglobin Combined with Electrospinning Composite Nanofibers. Anal. Lett..

[126] He P.W. (2021). Preparation of Bio-Phenol-Based Antibacterial Nanofiber Membranes and Their Application in Protective Masks.

[127] Kang L. (2022). Preparation and Filtration Performance of Electrospun Nanofiber Membranes for Protective Applications.

[128] Baselga-Lahoz M., Yus C., Arruebo M., Santamaria M.P., Ibarra M.R., Marco J.F. (2022). Submicronic Filtering Media Based on Electrospun Recycled PET Nanofibers: Development, Characterization and Method to Manufacture Surgical Masks. Nanomaterials.

[129] Elamri A., Zdiri K., Hamdaoui M., Baklouti S., Jridi M., Nasri M. (2023). Chitosan: A biopolymer for textile processes and products. Text. Res. J..

